# Functional metagenomics highlights varied infection states with dynamics of pathogens and antibiotic resistance in lower respiratory tract infections

**DOI:** 10.1016/j.heliyon.2024.e38380

**Published:** 2024-09-26

**Authors:** Uzma Shamim, Aanchal Yadav, Ranjeet Maurya, Priti Devi, Pallawi Kumari, Kanika, Kriti Khare, Bansidhar Tarai, Rajesh Pandey

**Affiliations:** a Division of Immunology and Infectious Disease Biology, INtegrative GENomics of HOst-PathogEn (INGEN-HOPE) laboratory, CSIR-Institute of Genomics and Integrative Biology (CSIR-IGIB), Mall Road, Delhi-110007, India; b Ashoka University, Sonipat, Haryana-131029, India; c Academy of Scientific and Innovative Research (AcSIR), Ghaziabad-201002, India; d Indraprastha Institute of Information Technology (IIIT), New Delhi-110020, India; e Max Super Speciality Hospital (A Unit of Devki Devi Foundation), Max Healthcare, Delhi 110017, India

**Keywords:** Lower respiratory tract Infection (LRTI), Pneumonia, Microbes, Antimicrobial resistance (AMR), Metagenomic next-generation sequencing (mNGS), RPIP, Explify, CZID seq

## Abstract

**Background:**

Antimicrobial resistance (AMR) amongst pathogenic bacterial species poses significant challenges in treating infections of the lower respiratory tract (LRT), leading to higher hospitalization and mortality rates.

**Methods:**

Bronchoalveolar lavage fluid (BALF) from 84 clinically adjudicated LRTI patients were subjected to respiratory pathogen ID/AMR (RPIP) enrichment and sequencing followed by Explify and CZID seq data analysis to identify potential LRTI pathogens and associated AMR genes. Patients were categorized as LRTI-WP (with pneumonia) and LRTI-WoP (without pneumonia).

**Findings:**

mNGS achieved 100 % pathogen detection compared to 73 % through clinician-used BioFire panel. Predominant pathogens included *Acinetobacter baumannii*, *Klebsiella pneumoniae* along with detection of *Aspergillus versicolor* and Herpes simplex virus. Double and polymicrobial infections were captured, involving non-respiratory pathogens like *Rothia mucilaginosa*, *Escherichia coli*, and *Moraxella osloensis*. AMR detection highlighted macrolide (MPH; ERM) and Sulfonamide (SUL) rich resistome in 60 % of patients followed by extended spectrum beta lactamase (OXA) and tetracycline (TET). LRTI-WP showed high abundance of *A. baumannii*, majorly associated with MPH whereas *K. pneumoniae* with beta-lactams was comparable in both groups. Differences in clinical severity may stem from non-respiratory pathogens, newly recognized via mNGS. CZID seq pipeline validated and revealed additional microbes and AMR genes in the cohort.

**Interpretation:**

The prevalence of common pathogens like *A. baumannii* and *K. pneumoniae* along with the non-respiratory pathogens identified by RPIP-Explify and CZID seq provided an understanding to evaluate the LRTI. mNGS is crucial for precise pathogen and antibiotic resistance detection, vital for combating antibiotic resistance.

## 1 Research in context

### 1.1 Evidence before this study

Lower Respiratory Tract Infections (LRTIs) impose significant burden worldwide, causing high rates of hospitalization and mortality, often leading to severe pneumonia. Misuse of antibiotics has fueled the rise of antimicrobial resistance (AMR) among LRTI-causing pathogens. Current diagnostic methods for LRTI primarily rely on culture and PCR-based tests, limiting pathogen detection and leading to broad-spectrum empirical antibiotic prescriptions in hospitals. These limitations prompted us to employ metagenomic next-generation sequencing (mNGS), offering unbiased detection of potential pathogens and AMR genes involved in the pathogenesis of LRTI but different phenotype of with and without pneumonia.

### 1.2 Added value of this study

We conducted a prospective study on 84 clinically adjudicated LRTI patients in India, admitted to hospital with severe respiratory discomfort. Our findings reveal the prevalence of common pathogens like *A. baumannii* and *K. pneumoniae* in LRTI alongside non-respiratory pathogens which could be identified only by culture-independent mNGS analysis methods, RPIP-Explify and CZID seq. The clinical severity (high ventilator support and mortality) of LRTI-WP patients could be assessed with elevated count of AMR genes captured in pneumonia-afflicted patients, when compared to those without pneumonia-like symptoms (LRTI-WoP).

### 1.3 Implications of all the available evidence

The BioFire panel, commonly utilized by clinicians for pathogen and AMR detection in LRTI patients, relies on the PCR method but lacks precision in diagnosis. Our research uncovered a spectrum of microbes, including non-respiratory bacteria, associated with LRTI. Additionally, we found that certain AMR classes, such as aminoglycosides, and ESBLs, not adequately detected by BioFire, demonstrated significant presence in WP and WoP cohorts, respectively. These mNGS-based findings, coupled with existing observations, offer potential for early pathogen and AMR gene detection, enhancing LRTI diagnosis and treatment efficacy.

## 2 Introduction

Lower Respiratory Tract Infections (LRTIs) have been a major burden in terms of mortality and morbidity rates all across the world accounting for approximately 2.6 million deaths in 2019, according to WHO [1]. These infections, if not controlled, could lead to severe pneumonia, septic shock and multiple organ dysfunctions in a subset of patients [2]. The etiological agents of LRTIs could be bacterial, viral or even fungal infections. Conventionally, empiric treatment of LRTIs, administered by medical practitioners relies on the results of sputum culture and smear wherein targeted antimicrobial medicines are prescribed [3].

However, inappropriate use of antibiotic drugs has increased precedence of antimicrobial resistance in causative LRTI pathogens whilst adding to low rates of pathogen isolation and sensitivity in their detection [4]. Antimicrobial Resistance (AMR) has become a major contributing factor for the increased mortality rates in LRTIs because of the inability of the antibiotics to inactivate the target pathogen in question [5]. Henceforth, the efficient detection of the broad range of pathogens along with their antimicrobial resistance patterns is imperative for defining the etiology of LRTIs. It can provide guidance to healthcare practitioners while treating cases requiring antibiotic therapy.

Current diagnostic tests for LRTI detection come with limitations. Although culture dependent diagnostic methods are the gold standard for pathogen detection, yet they have a low sensitivity along with labour intensive and time consuming [6]. Antimicrobial susceptibility testing (AST) for the detection of AMR is dependent on culture, which takes days for accurate detection of the AMR, delaying treatment procedures [7]. Highly accurate newer diagnostic tests also have their own limitations such as requirement of isolation from pure cultures in case of Microscopy matrix-assisted laser desorption ionization–time of flight mass spectrometry (MALDI-TOF), and inability to detect uncharacterized pathogens using 16S rRNA sequencing [6]. PCR and serology are other quick and convenient methods for LRTI diagnosis but limits the detection to a known breadth of pathogens [1]. The BioFire Pneumonia plus PCR based array is well documented for its usage in hospital/clinical settings over sputum culture (standard of care testing) yet it is designed to capture a common set of respiratory pathogens for pneumonia [[8], [9], [10]]. The shortcomings of LRTI diagnostic tests, results in application of broad-spectrum antibiotics as treatment regimens in hospital settings.

Applicability of metagenomics Next Generation Sequencing (mNGS) in infectious diseases have emerged as a superior diagnostic tool owing to their culture independency, detection of a broad range of pathogens along with the AMR genes associated with them, and identification of novel/rare microbial species implicated in LRTIs [6]. Recent advancements in mNGS technology have significantly enhanced its accuracy, speed, and utility in clinical diagnostics and research, enabling swift, effective and impartial information about the sequence of microbial nucleic acids, thus improving our knowledge of pathogen identification in the host-pathogen interaction domain [4]. Improvements include faster sequencing chemistries, sophisticated bioinformatics tools, and targeted enrichment techniques, which increase sensitivity and specificity. High-throughput and multiplexing capabilities, along with portable sequencing devices, have made mNGS more accessible and cost-effective [11]. LRTI diagnosis with NGS may provide insights for prescribing appropriate antibiotics and thus reducing the potential burden of treatment failure and of antibiotic resistance because of inappropriate antibiotic use [6]. Genomics-based AMR data has the potential to generate highly accurate predictions of the priority pathogen with anti-microbial resistance among the patients with infectious diseases. Integration with clinical workflows and other omics technologies, as well as the expansion of genomic databases, further enhances pathogen identification and AMR detection [12,13]. A comprehensive set of standard operating procedures (SOP) that would enable the use of NGS in clinical settings is currently lacking. These processes would include sample collection, amplification and library preparation, quality control, data analysis, and other related activities. SOPs for each step of detection and characterization of pathogenic organisms through NGS is therefore necessary to establish its increased usage in clinical diagnosis and to address important research questions [4].

In this study, we have performed RPIP (Respiratory Pathogen ID/AMR enrichment) based sequencing on 84 clinical LRTI cases. The Explify RPIP kit (Illumina® Inc, San Diego, CA, USA), a targeted NGS assay, enriches the detection of 282 pathogens (187 bacteria, 42 viruses, and 53 fungi) and AMR sequences from respiratory specimens, followed by a robust data analysis platform designed for the rapid and accurate prediction of resistance of 79 common respiratory pathogens to 26 drug classes based on the detection of >2000 associated AMR markers (https://www.illumina.com/content/dam/illumina/gcs/assembled-assets/marketing-literature/respiratory-pathogen-panel-table-m-gl-00115/respiratory-pathogen-id-amr-panel-table-m-gl-00115.pdf). The platform has been used in clinical and research settings, offering significant advantages in terms of speed, accuracy, and ease of use [[14], [15], [16]]. Deep clinical evaluation based on presenting symptoms and diagnosis, led us to phenotypically segregate the cases into LRTI and non-LRTI, and the LRTI cases further classified into LRTI with pneumonia (LRTI-WP) and LRTI without pneumonia (LRTI-WoP). Hospital based testing for microbes and AMR by the PCR-based BioFire pneumonia panel provided a reference point for precisely associating the clinical phenotype of LRTI with microbial abundance through mNGS. Additionally, the identified bacteria were analyzed for their resistance profile through AMR gene presence. We further conducted an integrative analysis to detect pathogens and AMR genes using an open-source, cloud-based metagenomics pipeline, CZID seq [17,18]. This bioinformatics platform is tailored particularly for identifying microbes and antimicrobial resistance markers from metagenomic datasets. Our comparative parallel analysis by CZID seq in turn highlighted the clinical utility of mNGS for respiratory infections and streamlining antibiotic usage based on AMR gene detection.

## 3 Methods

### 3.1 Ethics approval and consent to participate

The study was approved by the Institutional Ethics Committee of both CSIR-Institute of Genomics and Integrative Biology (CSIR-IGIB), and Max Super Speciality Hospital, under the approval number CSIR-IGIB/IHEC/2020-21/01. Written consent was taken from the participants for enrolment in the study.

### 3.2 Study design

The study was conducted with 84 BALF (Bronchoalveolar lavage fluid) samples of patients reporting to MAX Hospital, Delhi, India. A detailed clinical presentation and demographic data along with microbiology results from each patient's electronic medical record was collected and carefully documented for usage during analysis.

### 3.3 Analysis using the BioFire filmarray pneumonia panel

The BioFire FilmArray Pneumonia Panel pouch, which is a closed and disposable system, contains all the reagents required for nucleic acid extraction and purification, reverse transcription, and PCR. The stored specimens were analyzed using the PN panel. The BioFire FilmArray Pneumonia Panel (bioMérieux Japan Ltd.), has been approved by the Food and Drug Administration for the potential detection and identification of multiple respiratory viral and bacterial pathogens in addition to selected antimicrobial resistance genes from sputum or bronchoalveolar lavage (BAL)-like specimens from individuals with LRT infections. This assay includes targets for 18 bacteria and 08 viruses that commonly cause pneumonia as well as 07 antibiotic resistance genes (Additional File 1). Approximately 200, 50, or 10 μL of the BAL-like or sputum-like specimen was mixed with the sample buffer using a vortex mixer. After flashing in a centrifuge to remove the foam, the mixture was transferred to the sample injection vial and injected into the pouch via the pouch sample port. The pouch was scanned and loaded into the BioFire FilmArray 2.0, and the run was initiated. The BFPP includes two process controls in each pouch, which must both be positive for a run to pass. Runs that failed these internal process controls in our study were repeated with a new pouch. In the event of an invalid or indeterminate result, specimen testing was repeated once.

### 3.4 Nucleic acid isolation

Total nucleic acid (DNA/RNA) was extracted from bronchoalveolar lavage fluid (BALF) using the QIAsymphony SP and the QIAsymphony DSP Virus/Pathogen Midi Kit, Qiagen, following the manufacturer's recommendations. The elution was performed in 60 μL of buffer AVE. It is important to note that the QIAsymphony DSP Virus/Pathogen Kits are specifically designed for use in conjunction with the QIAsymphony SP instrument. These kits offer fully automated and simultaneous purification of viral nucleic acids and bacterial DNA. Leveraging magnetic-particle technology, the purification process ensures high-quality nucleic acids devoid of proteins, nucleases, and other impurities. The resulting purified nucleic acids are ready for direct utilization in downstream applications.

### 3.5 Library preparation and enrichment

Libraries for mNGS were prepared using the Illumina®/IDbyDNA Respiratory Pathogen ID/AMR Panel (RPIP) protocol and reagents from Illumina® Inc, San Diego, CA, USA. First, cDNA was synthesized from RNA and then combined with DNA in equal volumes. The library construction involved DNA tagmentation and adapter ligation, utilizing an Illumina® RNA Prep with enrichment kit (Illumina® Inc, San Diego, CA, USA). To enhance microbial content, libraries underwent overnight hybridization with RPIP probes. The captured libraries underwent 14 cycles of amplification, followed by cleaning with AMPure XP (Beckman Coulter). Quantification of libraries was performed using a Qubit dsDNA HS Assay kit (Thermo Fisher Scientific), and the fragment sizes of representative libraries were assessed with Agilent 2100 bioanalyzer. The enriched libraries were pooled to an equimolar concentration, normalized to 1 nM concentration, and further diluted to a loading concentration of 650 pM. Dual indexed paired-end sequencing was carried out using a high-output flow cell (600 cycles) on an Illumina NextSeq 2000 at 2 × 301 read length.

### 3.6 Detection of microbes and AMR genes

Analysis of sequencing data generated by the targeted RPIP workflow was accomplished using the automated metagenomics data analysis pipeline Explify RPIP and the cloud-based open-source bioinformatics platform Chan Zuckerberg ID (CZID or IDseq, v3.7, https://czid.org/). Raw sequencing files were uploaded to both platforms to identify microbes and AMR genes.

For the Explify Analysis Pipeline v4.26.1, we used the RPIP-5.11.6 panel database, which includes curated human, bacterial, viral, and fungal subsets from the NCBI nucleotide database and CARD (The Comprehensive Antibiotic Resistance Database) for AMR gene identification. The reference sequences were clustered to build the reference index and classified to taxids. The metagenomics classifier uses a k-mer based classification algorithm to classify each query sequence, usually a read, against a collection of reference sequences. The application delivers data analysis for simultaneous detection, quantification, and profiling of >280 pathogens and >2000 associated AMR markers accessed via Illumina BaseSpace. We can select individual samples or a Basespace Sequence Hub (BSSH) project folder containing samples for analysis. The application interface allows for read QC, filtered reporting for microorganisms and bacterial AMR markers. Each sample results are provided in a summary PDF report. Additionally, we can download an aggregate XLSX report for all samples in a BSSH analysis folder. All results included in the Explify reports were taken forward for result interpretation.

**Fig. 1:**
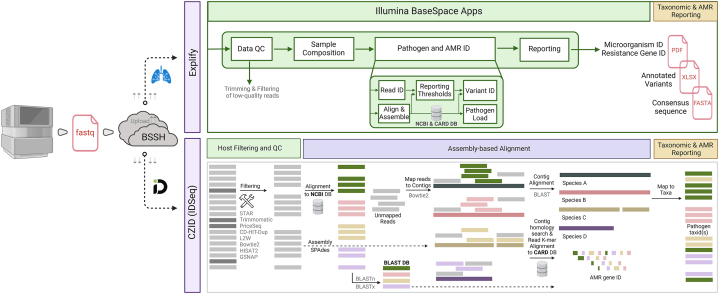
An overview of the Explify and IDseq pipelines data analysis workflow.

Similarly, raw FASTQ files were uploaded to the CZID portal, to identify microbes from the metagenomic data. The mNGS module and antimicrobial resistance pipeline was used for this purpose against background dataset of both nucleotide (NT) and protein (NR) NCBI databases along with Comprehensive Antibiotic Resistance Database (CARD) for AMR gene detection. The CZID pipeline involves several processing steps prior to analysis of non-host data, starting with alignment to the human genome (NCBI GRCh38) using STAR (v2.5.3) to remove the host sequences. As a result, these data files contain non-host sequences not only from pathogens but also from the environmental contaminants from skin and the laboratory, including bacteria, fungi, non-pathogenic viruses, and even vertebrates. Subsequent steps in the CZID pipeline (i.e., removal of low-quality reads, duplicate reads. low complexity reads, and additional human sequence filtering) further reduce contaminating and uninformative sequences from the non-human dataset. The remaining microbial reads then undergo an assembly-based alignment using an indexed version of the NCBI's GenBank (NT) database using GSNAP-L algorithm to identify the source of non-human sequences in the datasets and CARD (The Comprehensive Antibiotic Resistance Database) for AMR gene identification. We applied the following four threshold filters to determine the presence of a microbe in a sample: nucleotide (NT) Z-score ≥1, an average NT base pair alignment ≥50 base pairs, an E value ≥ 1e-10, and NT rPM ≥10 in the analysis. Nucleotide reads per million (NT rPM) was utilized for taxonomic identification in each sample and sorted by abundance, independently for each sample. Both the CZID platform and the Explify RPIP data analysis platforms read QC which includes trimming and filtering steps of removal of low-quality reads, duplicate reads. low complexity reads, and additional human sequence filtering. Fig. 1 describes an overview of the Explify and IDseq pipeline steps and data analysis workflow.

## 4 Statistics and data analysis

### 4.1 Correlation analysis and visualization

Pairwise Pearson correlations were generated using Pearson's correlations coefficient value between microbial species and Antimicrobial resistant genes (AMR) calculated via the rcorr function in the Hmisc (https://cran.r-project.org/web/packages/Hmisc/index.html) (Harrell Jr, F.E. and Harrell Jr, M.F.E., 2019. Package ‘hmisc’. CRAN2018, 2019, pp.235-236.) R package. To visualize the correlation, corrplot R package (https://cran.r-project.org/web/packages/corrplot/vignettes/corrplot-intro.html) (Wei, T., Simko, V., Levy, M., Xie, Y., Jin, Y. and Zemla, J., 2017. Package ‘corrplot’. Statistician, 56 (316), p.e24.) was used. Blue and red colors indicate positive and negative correlation, respectively. The color density, circle size, and numbers reflect the scale of correlation. ∗Significant level (∗p < 0.05; ∗∗p < 0.01; ∗∗∗p < 0.001).

### 4.2 Heatmap and network analysis

The microbial species present in >10 % of samples across the distinct groups were displayed together in a heatmap created in python (version 3.9.7) with seaborn library (version 0.11.2). The groups are color-coded for relative values as LRTI-WP in orange, LRTI-WoP in blue, Non-LRTI in green, and No-CD in purple. Further, the bacterial species and their associated AMR gene families in WP and WoP groups were showed using a gene-microbe network. The network was created in Cytoscape (Version: 3.9.1), where blue nodes represent LRTI-WoP and orange nodes represent LRTI-WP group. Large nodes represent significant microbes and edges are represented in red (negative) and blue (positive) correlation values.

### 4.3 Role of funders

The funders had no role in the study design, data collection, data analyses, interpretation or writing of the report.

## 5 Results

### 5.1 Patient cohort and study strategy

**Fig. 2:**
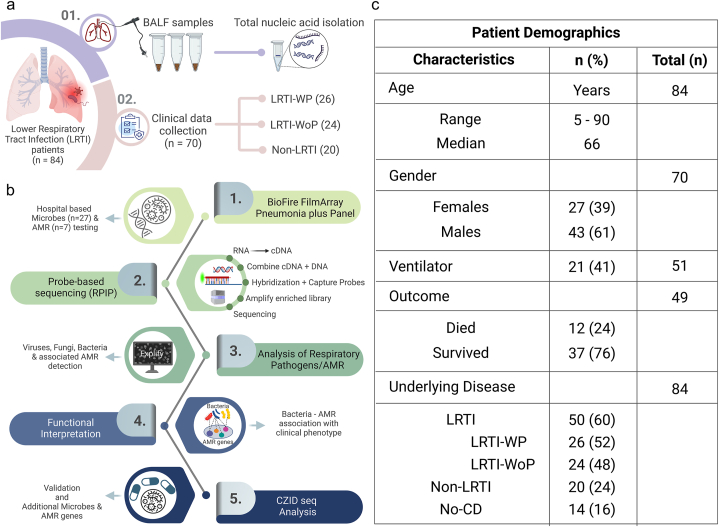
**Overview of the study design, patients' characteristics and investigation methodologies. a)** Schematic illustration depicting collection of BALF samples from patients with LRTI and isolation of total nucleic acids. Segregation of patients based on clinical details into LRTI with Pneumonia (LRTI-WP), LRTI without Pneumonia (LRTI-WoP), and Non-LRTI groups. **b)** Graphical representation outlined the multi-layered workflow of the experimental process. **c)** Patient demographics and broad clinical characteristics, including missing data information.

This study comprised of patients (n = 84) presenting to the hospital with symptoms of severe respiratory discomfort, which led them to be suspected of lower respiratory tract infections (LRTI) or clinically adjudicated LRTI. At the hospital, BALF (Bronchoalveolar lavage fluid) specimens were collected from the patients and total nucleic acid was extracted for the assessment of microbial presence and related AMR genes through the BioFire FilmArray Pneumonia Plus panel. Demographic and detailed clinical presentation was collected from the patients while admission. The patients were segregated into two groups, LRTI with Pneumonia (LRTI-WP; n = 26) and LRTI without Pneumonia (LRTI-WoP, n = 24). The diagnosis of Pneumonia was based on combination of laboratory (TLC), radiological (X-ray, CT chest) and microbiological findings (culture of urine, blood, Sputum, BAL) (Pneumonia (Ventilator-associated [VAP] and non-ventilator- associated Pneumonia [PNEU]) Event). There were few patients (n = 20), which did not fall in either category based on diagnosis, as they were related to a variety of non-infectious syndromes, hence they were assigned to another group, named “Non-LRTI”. The clinical details were missing for another set of 14 patients referred to as “No CD” (Fig. 2a). The clinical information of the patients received from the hospital included presenting complaint, diagnosis, requirement of ventilator support, medicines prescribed, laboratory tests performed and outcome, provided as **Additional File 2**.

Fig. 2b elaborates the study design highlighting several layers of investigation for a comprehensive understanding of microbes leading to LRTI and the type of AMR they carry that might worsen respiratory infections. The BioFire data, which uses PCR based detection of microbes and AMR in hospital settings, was used as the first line of investigation in this study.

While the BioFire PCR-based method is highly effective for rapid pathogen detection (reported sensitivity 96.2 % and specificity 98.3 %), it may have limitations in identifying the limited pathogens and AMR markers included in the target panel. Therefore, an mNGS-based hybridization-capture-sequencing workflow (RPIP; Respiratory Pathogen ID/AMR Enrichment Panel Kit/probe-based sequencing) was carried out for all 84 BALF samples. This approach aids in detecting a broader range of pathogens, including those present in very low quantities, by enriching microbial DNA with targeted probes. Data analysis by Explify curated the microbes and AMR genes captured across the samples, classifying 93.8 % of reads to the targeted capture probes, 5.7 % to those that are not targeted, 0.5 % ambiguous reads that cannot be assigned to one category. A detailed comparison is given in Table 1. We performed a parallel CZID seq analysis using the same sequence generated data, for comparative analysis. Finally, collating all data, the microbes, associated resistance genes, and major AMR classes were identified across the groups, which provided functional relevance to contemplate the antibiotic treatment regimen used to treat LRTI in hospital settings.

**Table 1:** A detailed comparison between BioFire and RPIP methods.

Features	BioFire (PCR-Based) Method	RPIP (Probe-Based) NGS Method
Detection Technology	PCR-based	Probe-based NGS
Hands-on time	2 min	<2 h
Pathogen Range	Targets 18 bacteria, 8 viruses, and 7 antibiotic resistance genes	(180+ bacteria, 50+ fungi, and 40+ viruses, including SARS-CoV-2) and antimicrobial resistance alleles (2000+).
Comprehensive genome coverage		SARS-CoV-2 and Influenza A/B viruses.
Data Analysis Platform		Automated Explify RPIP Data Analysis platform using Illumina BaseSpace.
Enrichment Technique		Uses overnight hybridization with RPIP probes to enhance microbial content.
Limitations	May not identify novel or unexpected pathogens not included in the target panel.	Requires more complex and time-consuming analysis, higher cost.
Application	Rapid and comprehensive detection of known respiratory pathogens.	Detailed and extensive profiling of microbial communities and resistance markers.

The patient demographics and broad clinical characteristics are highlighted in Fig. 2c along with missing data information. The median age of the patients was 66 years reflecting a general trend of increased respiratory infections in the higher age groups. Percentage of male patients was higher (61 %) than females (39 %). Nearly half of the patients (41 %) required ventilator support. The overall survival rate was high with 76 % of patients recovering from LRTI infections. The categorization of the patients’ underlying disease status based on diagnosis is also mentioned.

### 5.2 Comprehensive functional evaluation of hospital acquired data

#### 5.2.1 Clinical interpretation of patient reports highlight diversity in LRTI disease phenotype

**Fig. 3:**
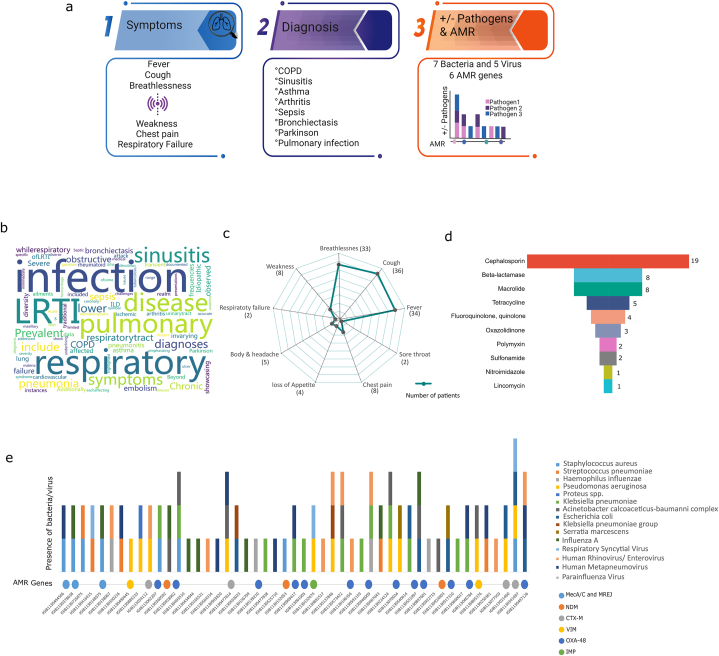
**Diversity of microbes and antimicrobial resistance (AMR) genes in LRTI patients using PCR-based BioFire panel a)** Schematic outline for major clinical factors including symptoms, diagnosis, and infecting microbial species or captured AMR genes observed across the patient cohort. **b)** Word cloud characterizes the diagnoses associated with LRTI. **c)** Spider plot displays the diversity of symptoms exhibited by LRTI patients. **d)** Inverted pyramid illustrates the distribution of 10 antibiotic classes prescribed to the LRTI patients. **e)** Stacked bar plot depicts the microbes (bacteria and viruses) captured by the BioFire panel and their associated AMR genes in BALF specimens.

Clinically adjudicated LRTI prompts initiation of broad-spectrum empirical antimicrobial therapy in clinical settings. Importantly, the microbial species leading to infection needs identification that can potentially guide downstream treatment regimen in future with more scientific evidence, especially the selection of antibiotics in the hospital settings. Hence, as a first investigation, we tried to outline the major clinical factors that were taken into consideration, like presenting symptoms, diagnosis and infecting microbial species/captured AMR genes across the patient cohort in the hospital (Fig. 3a). The symptoms maximally found in the patients were that of breathlessness with varying degrees/days of fever and cough. Few patients also reported chest pain and weakness (Fig. 3b). The clinical diagnosis was essentially that of LRTI, Pneumonia (interstitial, organizing, atypical, bronchopneumonia), few cases of Sinusitis, Asthma, COPD Bronchiectasis, Respiratory failure and Septic shock. Patients were also diagnosed with other underlying comorbid conditions like cardiovascular disease, acute/chronic kidney disease apart from hypertension and diabetes (Fig. 3c). Notably, all LRTI patients did not show pneumonia in the diagnosis, which led us to classify them as LRTI-WP and LRTI-WoP. Moreover, 20 patients were categorized as non-LRTI as their diagnosis involved other illness, such as Dengue like illness, Jaundice, Melena, Wernicke Encephalopathy with Septicemia, Uremic Lung, Tuberculosis, Acute and Severe Necrotizing Pancreatitis.

#### 5.2.2 BioFire generated microbes and AMR genes partially account for the antimicrobial treatment regimens to the LRTI patients

The BioFire panel captured microbes in the BALF specimens of 51 (73 %) patients out of 70, whose clinical referrals were available (Fig. 3e). 08 bacteria and 05 viruses were identified in the patient cohort, wherein, a combination of both viruses/bacteria and bacteria only were present in multiple samples along with singularly occurring species. 24 patients harboured one bacterial species, whilst 15 reported 02 and only 02 patients reported presence of 03 bacterial species. The prevalence of the 08 bacterial species in patients were *Klebsiella pneumoniae* (15), *Pseudomonas aeruginosa* (12), *Acinetobacter calcoaceticus-baumannii* complex (9), *Staphylococcus aureus* (08), *Streptococcus pneumoniae* (05), *Haemophilus influenzae* (04), *Escherichia coli* (04) with *Serratia marcescens*, and *Proteus* spp. present in 02 and 01 samples. Human Metapneumovirus and Human Rhinovirus/Enterovirus were present in 10 samples each, with 01 patient reporting both. Notably, 21 and 11 patients carried either bacterial or viral species whereas 19 patients carried both viral and bacterial infection. Looking at the AMR genes across the 40 patients reporting bacterial infection, 26 of them showed presence of AMR genes (Fig. 3e). Methicillin-resistant *Staphylococcus aureus* (MRSA) was detected in 4/8 patients. The other AMR genes detected were CTX-M (17) [extended spectrum beta lactamase], NDM (17), VIM (4), OXA-48 (12) and IMP (1) [carbapenemase]. The antibiotics prescribed to the patients are illustrated in Fig. 3d where majorly beta lactams of subclass cephalosporin (18) were given. Cephalosporin was either given alone (9) or in combination with macrolide or tetracycline. Carbapenem and macrolide were also given singularly to 06 and 05 patients respectively. Few patients received Oxazolidinone, Fluoroquinolone, Sulfonamide and glycopeptide. Antibiotic information was missing for 22 patients.

#### 5.2.3 RPIP-Explify demonstrates potential role of *Acinetobacter baumannii* and *Klebsiella pneumoniae* as major resistant microbial population in the LRTI patients

**Fig. 4a:**
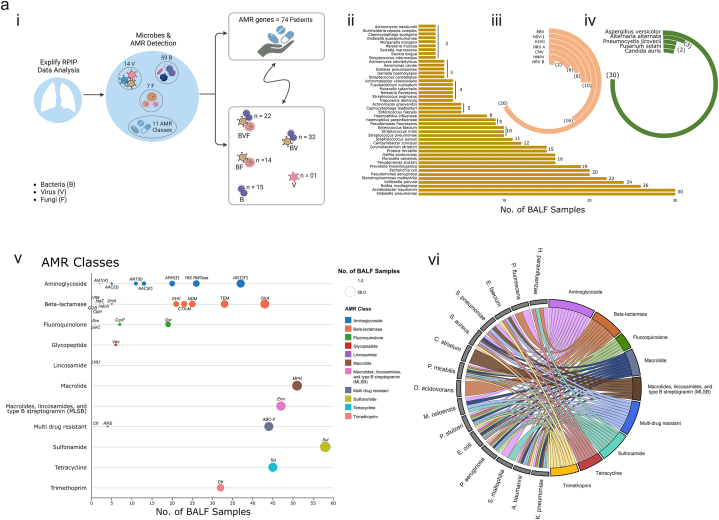

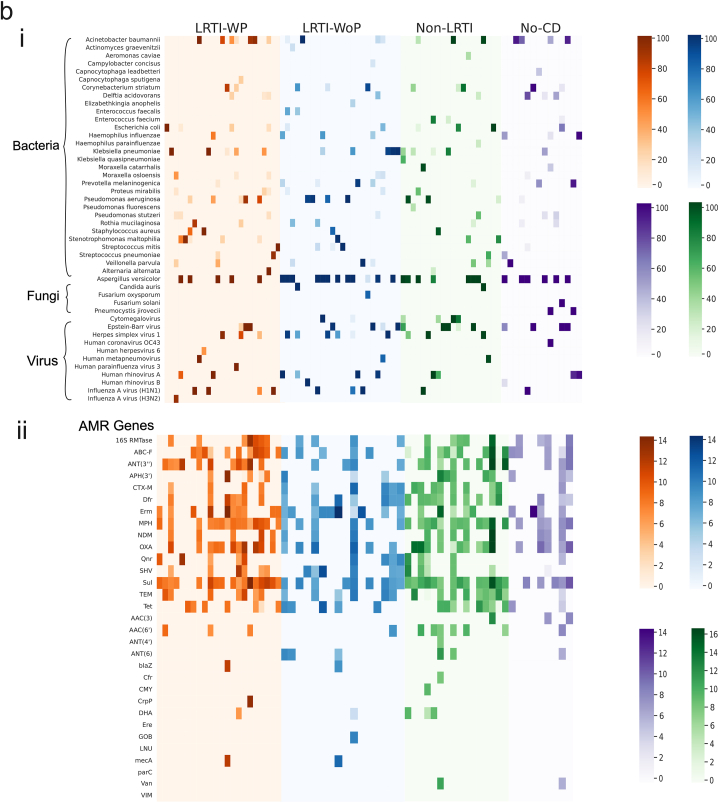
Fig. 4a:Demonstrates the microbial profile and associated AMR gene families using the RPIP-Explify mNGS pipeline. i) Graphic overview denoting identification of 59 bacteria, 14 viruses, and 07 fungi along with 11 AMR classes across 84 patient samples. **ii)** Horizontal bar plot displays the bacteria species. Radial bar plot depicts the presence of **iii)** viruses, **iv)** fungi, each indicating their occurrence in a different number of LRTI samples. **v)** Dot plot exhibits 11 AMR classes detected in the samples, with the size of each dot corresponding to its presence in a specific number of samples. **vi)** Circos plot represents the association of AMR classes with different bacteria detected in the patient cohort. Fig. 4b: Overview of the microbial profile and associated AMR genes. Heatmap displayed the prevalence of **i)** bacteria, viruses, and fungi; **ii)** AMR gene distribution across different patient groups, categorized as LRTI-WP, LRTI-WoP, Non-LRTI, and No CD.

Clinically adjudicated LRTI manifested in different phenotypes such as LRTI-WP, LRTI-WoP and non-LRTI. In order to understand the clinical phenotype diversity of LRTI, we performed mNGS using the Illumina RPIP Panel Kit. Metagenomic analysis by RPIP-Explify led to the detection of 59 bacteria, 14 viruses and 07 fungi across all 84 patient samples signifying 100 % detection rate (Fig. 4a–i). Removing those microbial species that demonstrated presence in only 01 sample, we retained 44 bacteria, 08 viruses and 05 fungi across the samples. We identified multiple combinations of microbial species across samples. Bacteria (B), virus (V) and fungi (F) were present in 22 samples whereas BV and BF combinations were present in 32 and 14 samples respectively. Presence of only bacterial species were in 15 whilst only viral species in 01 sample. Importantly, LRT disease-causing pathogens *Klebsiella pneumoniae* and *Acinetobacter baumannii* were maximally present in 30/84 samples (35 %), whilst others like *Pseudomonas aeruginosa* (20), *Staphylococcus aureus* (11) and *Streptococcus pneumoniae* (10) were displayed in lesser number of samples. The other species present in >10 % of samples were varied, with few as part of normal flora of respiratory tract which may show association with disease like *Rothia mucilaginosa* (26), *Veillonella parvula* (24), *Prevotella melaninogenica* (19), *Moraxella osloensis* (16) *Corynebacterium striatum* (15), *Streptococcus mitis* (10) and *Haemophilus parainfluenzae* (09) whilst others of gastro-intestinal flora like *Escherichia coli* (20), *Proteus mirabilis* (15), *Campylobacter concisus* (12) and *Enterococcus faecium* (10). Few were environmental inhabitants but reported disease associations like *Stenotrophomonas maltophilia* (22), *Pseudomonas stutzeri* (16), *Delftia acidovorans* (16) and *Pseudomonas fluorescens* (09) (Fig. 4a–ii). The viral species maximally identified in patients were Epstein Barr (EBV) and Herpes simplex virus (HSV) in 26 and 16 patients respectively. Human Rhinovirus (HRV), Cytomegalovirus (CMV) and Human metapneumovirus (HMPV) were also detected in variable numbers (Fig. 4a–iii). The fungi *Aspergillus versicolor* infected 30 patients (Fig. 4a–iv). The Explify analysis also detected AMR genes across 74 samples wherein 10 samples did not report any AMR gene. SUL gene belonging to Sulfonamide class was detected in 78 % (58/74) of patients, followed by MPH (macrolide; n = 51), ERM (MLSb; n = 47), TET (tetracycline; n = 45) and OXA (class D carbapenem; n = 43). Interestingly, along with OXA, carbapenem associated AMR genes and ESBLs like NDM, SHV, TEM, CTX-M were commonly present in more than 25 % of samples (Fig. 4a–v). Similarly, 16s RTMases and ANT (3″) providing resistance to Aminoglycosides and ABC-F to multiple antibiotic classes, also demonstrated an increasing trend in the LRTI cohort (Fig. 4a–v). We also tried to extrapolate the detection of AMR genes corresponding to different bacterial species, captured in the LRTI patients. These AMR genes are reportedly detected for several bacterial species. The circos plot defines all the AMR genes (clubbed into their respective classes) associated with different bacteria that were detected in the patient cohort. *A. baumannii*, *K. pneumoniae*, *P. aeruginosa*, *E. coli*, *S. aureus* and *P. mirabilis* showed association with many AMR classes, whereas *C. striatum* was linked to MLSb, *D. acidovorans* with beta-lactamase and *S. maltophilia* with aminoglycosides (Fig. 4a–vi).

The prevalence of microbial species (BVF) across different patients’ groups is collated as a heatmap, taking only those species which are present in >10 % composition in each sample (Fig. 4b–i; Additional File 3). A similar visualization of AMR genes contributed by these microbial species and prevalent across different patient groups is illustrated in Fig. 4b–ii.

#### 5.2.4 Higher association of pathogenic microbes with diverse AMR gene families explains the clinical LRTI-WP and LRTI-WoP diagnosis to an extent

Next, we delved into understanding the microbial population comprising the resistome in the LRTI patients and its association with the observed phenotype diversity. Since, LRTI is caused by perturbation of the airway microbiome that percolates into the lungs, polymicrobial population was captured in the clinically adjudicated patients. Dysbiosis causes predominance of pathogenic bacteria which may be potentially causal for the phenotype. Hence, we considered those microbes for downstream analysis which were >10 % in composition, amongst all identified microbes present in each patient. Notably, as seen in the heatmap (Fig. 4b), both LRTI as well as non-LRTI patients, contained pathogenic species known to cause severe respiratory infections in humans majorly, *A. baumannii*, *K. pneumoniae*, *S. pneumoniae* and *P. aeruginosa*. Other microbes like *S. maltophilia*, *E. coli*, *C. striatum*, *P. mirabilis*, *P. melaninogenica*, *V. parvula*, *P. stutzeri* and *D. acidovorans* were also detected across multiple patients but essentially in the LRTI group, with the exception of *E. coli* which inhabited both the patient groups. Different species of *Moraxella* and *Enterococcus* inhabited patients of LRTI, *M. osloensis*; *E. faecalis* and non-LRTI, *M. catarrhalis*; *E. faecium*. Importantly, the patients in non-LRTI group diagnosed with pulmonary TB showed the presence of *Mycobacterium tuberculosis* complex and Rhinovirus. The fungal species identified across the two groups were the same, but there was dominance of viral species in LRTI, which was not observed in non-LRTI. Reasonably, although the clinical diagnosis for non-LRTI patients were different, yet based on the microbial profile of BALF, it can be professed that infection by conventional pathogenic species were redundantly found in LRTI as well as non-LRTI patients highlighting need for more studies going forward in different study cohorts, especially with county as big and populous as India (**Additional File 4**).

**Fig. 5:**
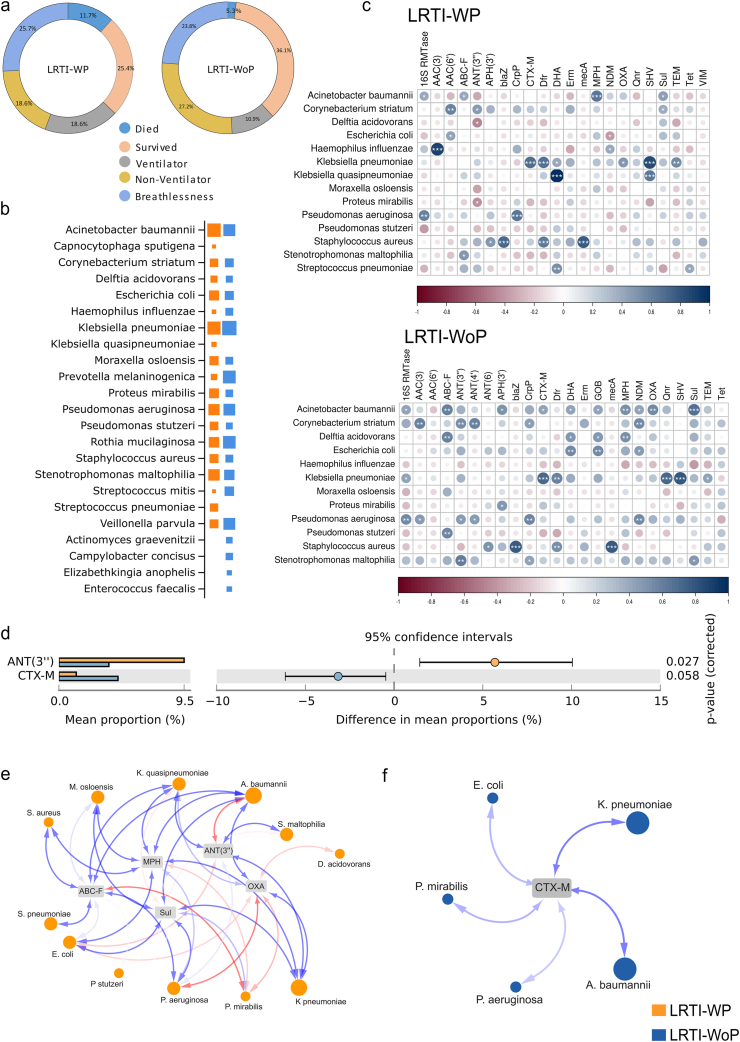
**Microbes and AMR genes potentially associated with LRTI-WP and LRTI-WoP**. **a)** Comparison of clinical parameters: survival outcome, ventilator requirement, presence of breathlessness. **b)** Plot represents common and unique bacterial species based on normalized read counts. **c)** Correlation plot shows statistically significant associations between detected bacterial species and AMR genes. **d)** STAMP analysis identifies the significant AMR genes association with WP and WoP. **e)** Network depicts the association patterns between bacteria and their associated AMR genes.

Interestingly, the LRTI manifested with pneumonia in 26 patients whereas the remaining 24 patients did not feature pneumonia at the time of bronchoscopy and BALF sample collection. Moreover, hospital stay for LRTI-WP demonstrated the requirement of ventilator support for a higher number of patients with increased mortality rate, depicting clinical severity in patients with pneumonia (Fig. 5a). A total of 19 and 20 bacterial species were detected in WP and WoP respectively (>10 % in composition) with 16 common species between the two groups (Fig. 5b). Single infection, where the composition of one bacterial species was abnormally high >80 %, was identified with *A. baumannii* (3), *K. pneumoniae* (2) and single cases of *S. aureus*, *S. pneumonia*, *S. mitis*, *S. maltophilia*, *P. aeruginosa*, *E. coli* and *R. mucilaginosa* in the LRTI-WP group. Similarly, the other patients demonstrated either double (5) or multiple (9) bacterial species with variable composition between 10 and 80 %. Similarly, LRTI-WoP group also displayed 12 cases of single infections with *K. pneumoniae* (3) and *P. aeruginosa* (3) whilst single cases with *S. mitis*, *V. parvula*, *P. melaninogenica*, *A. baumannii*, *C. striatum* and *S. maltophilia*. Double and multiple microbial presence was detected in 06 cases each in the WoP group. Notably, *A. baumannii* infection was more prevalent in the WP group with 07 patients whilst 04 patients in the WoP. Contrarily, *K. pneumoniae* was comparable with 05 cases in both WP and WoP groups. LRTI-WP also reported *S. maltophilia* in 05 cases. Moreover, the second bacteria present in the double infections in LRTI-WoP were largely species less likely to cause severe respiratory disease like *Streptococcus mitis*, *Enterococcus faecalis*, *Elizabethkingia anopheles* and *Prevotella melaninogenica*. The unique species identified in the WP group were *Streptococcus pneumoniae* (single infection), *Klebsiella quasipneumoniae*; *Haemophilus parainfluenzae*, (both double infection) and *Capnocytophaga sputigena* (multiple infection). In WoP group, *Elizabethkingia anopheles* (double infection), whilst *Actinomyces graevenitzii*, *Campylobacter concisus* and *Enterococcus faecalis* were present in the polymicrobial infection cases (Table 2).

**Table 2:** Bacterial species in mono- and poly-microbial infections in LRTI-WP and LRTI-WoP patients.

Type of infection	LRTI-WP (26)		LRTI-WoP (24)	
	n (%)	Bacteria	n (%)	Bacteria
**Single infection**	12 (46.15 %)	*A. baumannii* (3); *K. pneumoniae* (2); *S. aureus*; *S. pneumoniae*; *S. mitis*; *S. maltophilia*; *E. coli*; *P. aeruginosa*; *R. mucilaginosa*	12 (50 %)	*P. aeruginosa* (3); *K. pneumoniae* (3); *S. mitis*; *V. parvula*; *P. melaninogenica*; *A. baumannii*; *C. striatum*; *S. maltophilia*
**Double infection**	5 (19.23 %)		6 (25 %)	
	Case I	*P. aeruginosa*, *H. influenzae*		*P. aeruginosa*, *C. striatum*
	Case 2	*S. maltophilia*, *A. baumannii*		*S. aureu*s, *S. mitis*
	Case 3	*C. striatum*, *K. pneumoniae*		*H. influenzae*, *K. pneumoniae*
	Case 4	*K. pneumoniae*, *K. quasipneumoniae*		*C. striatum*, *E. faecalis*
	Case 5	*P. aeruginosa*, *S. maltophilia*		*K. pneumoniae*, *E. anophelis*
	Case 6			*H. influenzae*, *P. melaninogenica*
**polymicrobial infection**	9 (34.61 %)		6 (25 %)	
	Case I	*R. mucilaginosa*, *P. aeruginosa*, *C. sputigena*		*P. melaninogenica*, *R. mucilaginosa*, *S. mitis*
	Case 2	*P. aeruginosa*, *S. aureus*, *S. maltophilia*		*C. concisus*, *H. influenzae*, *P. melaninogenica*, *V. parvula*
	Case 3	*P. melaninogenica*, *S. pneumoniae*, *V. parvula*		*A. baumannii*, *D. acidovorans*, *M. osloensis*, *P. stutzeri*
	Case 4	*A. baumannii*, *D. acidovorans*, *E. coli*		*A. graevenitzii*, *R. mucilaginosa*, *V. parvula*
	Case 5	*M. osloensis*, *P. mirabilis*, *P. stutzeri*, *V. parvula*		*A. baumannii*, *E. coli*, *M.osloensis*, *P. mirabilis*
	Case 6	*A. baumannii*, *C. striatum*, *K. pneumoniae*, *M. osloensis*, *P. mirabilis*		*A. baumannii*, *E. faecalis*, *E. coli*, *P. mirabilis*
	Case 7	*A. baumannii*, *D. acidovorans*, *E. coli*, *M. osloensis*, *P. mirabilis*, *P. stutzeri*		
	Case 8	*E. coli*, *P. stutzeri*, *S. maltophilia*		
	Case 9	*D. acidovorans*, *K. pneumoniae*, *P. mirabilis*, *P. stutzeri*		

Next, we looked into the AMR genes detected in the WP and WoP groups corresponding to the bacterial species prevalent in each group. 25 AMR gene families were identified in both the groups, wherein 21 genes were commonly present in both groups. Strikingly, SUL, MPH, OXA, ABC-F and ANT (3″) genes were present in higher number of WP patients, when compared with the WoP patients. Notably, CTX-M dominated WoP group. Furthermore, aminoglycosides ANT4″ and ANT6″ were uniquely present in the WoP whereas VIM and GOB beta lactams were uniquely present in WP and WoP, respectively (Table 3). Subsequently, we performed correlation analysis between the detected bacterial species and AMR genes to identify the statistically significant AMR genes coming from the bacterial species in both the groups. *A. baumannii* majorly provides MPH (macrolide resistance) in the WP group. The other minor associations of *A. baumannii* have been traced to SUL, ABC-F and 16S rRNA methylases (RMTases) genes. *K. pneumoniae* is seen providing resistance with SHV, OXA, CTX-M, and TEM (beta-lactams) genes (Fig. 5c). In WoP patients, CTX-M resistance is majorly provided by *K. pneumoniae*. It also shows high association with SHV, and Qnr. *P. aeruginosa* is significantly correlated with 16S RMTase, NDM and CrP (Fig. 5c). Interestingly, along with MecA, *S. aureus* showed significant association with blaZ and Dfr in both the groups. We also performed STAMP analysis between WP and WoP groups to identify discrete AMR genes for the two groups. Corroborating with the above correlation analysis, we got ANT (3″) as a significant AMR gene in WP while CTX-M for the WoP group (Fig. 5d). Along with the true associations depicted by correlation plots, where the bacteria are reported to carry those AMR genes, it also showed associations wherein the AMR genes have not been shown to be harboured by the bacteria. Henceforth, we plotted the AMR-bacteria networks to capture the association patterns between them. For the WP group, along with ANT (3″), we analyzed the associations with other AMR genes contributing to resistance in a higher number of WP patients such as MPH, OXA, SUL and ABC-F. *A. baumannii* and *K. pneumoniae* showed positive correlations with multiple AMR genes. Several other bacteria like *S. maltophilia, P. aeruginosa*, *E. coli* and *Klebsiella quasipneumoniae* were also observed for associations with ANT (3”), SUL and ABC-F (Fig. 5e). In WoP, CTX-M showed positive correlation with *A. baumannii* and *K. pneumoniae* only (Fig. 5f).

**Table 3:** Presence of AMR gene families associated with the bacterial species detected in LRTI-WP and LRTI-WoP patients.

AMR Gene Family		Counts (n)	
		WP	WoP
ABC-F ATP-binding cassette ribosomal protection protein	ABC-F	**12**	**6**
ANT (3″)	ANT (3″)	**12**	**5**
16S rRNA methyltransferase (G1405)	16S RMTase	8	5
APH(3′)	APH(3′)	6	4
AAC (6′)	AAC (6′)	3	1
AAC (3)	AAC (3)	1	1
ANT (4)	ANT (4)	0	1
ANT (6)	ANT (6)	0	3
macrolide phosphotransferase (MPH)	MPH	**16**	**7**
sulfonamide resistant sul	Sul	**18**	**10**
OXA beta-lactamase	OXA	**12**	**7**
CTX-M beta-lactamase	CTX-M	**4**	**7**
NDM beta-lactamase	NDM	7	4
TEM beta-lactamase	TEM	7	6
SHV beta-lactamase	SHV	6	6
blaZ beta-lactamase	blaZ	1	1
DHA beta-lactamase	DHA	1	1
VIM beta-lactamase	VIM	1	0
GOB beta-lactamase	GOB	0	1
methicillin resistant PBP2	mecA	1	1
ciprofloxacin phosphotransferase	CrpP	2	1
trimethoprim resistant dihydrofolate reductase	Dfr	7	7
quinolone resistance protein	Qnr	6	5
Erm	Erm	11	9
tetracycline-resistant ribosomal protection protein	Tet	7	5

### 5.3 Microbial and AMR profiling using the CZID pipeline provides validation and extensive AMR gene information

**Fig. 6:**
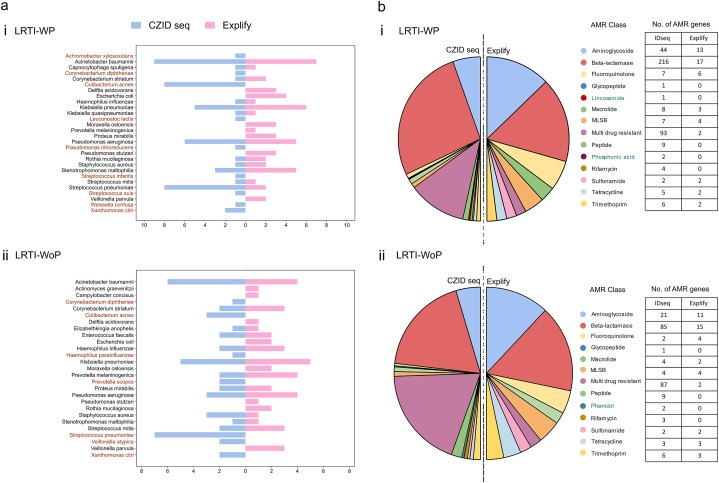
**Microbes and associated AMR classes detected using CZID seq and compared with Explify-RPIP. a)** Comparison of bacterial composition obtained using the 2 mNGS pipelines for (i) LRTI-WP and (ii) LRTI-WoP groups. Brown color indicates the unique microbes obtained by CZID seq. **b)** The pie chart illustrates the total number of AMR genes obtained in the AMR class in (i) LRTI-WP and (ii) LRTI-WoP group from CZID seq and RPIP-Explify pipeline. The teal color indicates the unique AMR class of the respective group. (For interpretation of the references to color in this figure legend, the reader is referred to the Web version of this article.)

We employed the CZID seq pipeline to examine the resistance gene landscape within our mNGS data. CZID seq is particularly adept at detecting divergent viruses and human pathogens. Leveraging the comprehensive NCBI NT and NR databases, CZID seq demonstrates strong performance at novel microbe identification. Across the WP and WoP patients, the CZID seq NT database exhibited enhanced specificity while identifying 21 and 19 bacterial species respectively, falling in >10 % composition, with varied presentation majorly as double and polymicrobial infection. Comparison with RPIP-Explify results revealed the consistent detection of the known pathogenic species, *A. baumannii, K. pneumoniae*, *P. aeruginosa*, *S. pneumonia*, *S. aureus* and *H. influenza* in both the subgroup of patients, mirroring the Explify findings. Notably, our analysis using CZID seq revealed the presence of *A. xylosoxidans*, an opportunistic bacterium known to cause infections in immunocompromised patients, with a high mortality rate within the WP patients. Interestingly, this finding was not observed when examining species with abundance greater than 10 % using Explify. Additionally, along with lesser-associated microbes of LRTI captured through Explify (*S. maltophilia*, *C. straitum* and *S. mitis*), we also identified *C. diphtheria*, *C. acnes* and *X. citri* in both the groups through CZID seq analysis. Interestingly, CZID seq detected *S. pneumoniae* in higher number of samples in both the WP and WoP patients, although its presence was essentially in double or polymicrobial infection states with percent composition ranging between 10 and 60 %. Fig. 6a demonstrates the common and unique microbes across WP (**i**) and WoP (**ii**) detected by Explify and CZID seq. *P. nitroreducens*, *S. infantis*, *S. suis*, *W. confuse* and *L. lactis* are the unique species identified in WP whereas *V. atypica* and *P. scopus* in WoP patients.

Among the bacteria present at more than 10 % abundance, nine AMR classes were concurrently identified by both the Explify and CZID seq bioinformatics suite. Notably, IDseq revealed an additional of five classes in the WP (Fig. 6b–i) and four classes in the WoP (Fig. 6b–ii) patients. Specifically, in the WP group, this included Rifamycin, Phosphonic acid, Peptide, Lincosamide, and Glycopeptide, while in the WoP group, they encompassed Rifamycin, Phenicol, Peptide, and Glycopeptide. Unique presence was observed for certain classes: Lincosamide and Phosphonic acid in WP, and Phenicol in WoP, each with a small number of associated genes.

A total of 405 AMR genes were detected in the WP group, significantly surpassing the 229 genes found in the WoP. Notably, beyond the findings of the Explify analysis, multiple genes conferring resistance to beta-lactams were identified, including those from the CMY, SHW, VIM, CME, L1 family and KPC beta-lactamase families, present in both LRTI groups. Detailed information regarding the AMR classes, families, and their respective genes is provided in the **Additional File 5**.

## 6 Discussion

Lower respiratory tract infections (LRTIs) comprise a diverse range of diseases, such as pneumonia, chronic obstructive pulmonary disease (COPD), bronchitis, and other conditions affecting the lower airways. These infections pose a significant public health concern, accounting for millions of hospitalizations with substantial mortality rates [19]. Various common bacteria, such as *Streptococcus pneumoniae*, *Klebsiella pneumoniae*, and *Haemophilus influenzae*, along with other opportunistic pathogens, such as respiratory viruses, and fungi are reported to cause LRTIs [20]. The management of LRTIs faces a substantial obstacle due to the rise of multidrug-resistant strains and the rapid spread of antibiotic resistance. The economist Jim O'Neill's 2016 report projected a potential surge in annual deaths due to Antimicrobial Resistance (AMR) to 10 million by 2050, highlighting the critical need for global AMR surveillance.

This study was an attempt to explore the comprehensive utility of mNGS in LRTI cases, wherein it focuses on the enrichment of specific common respiratory microbial species and AMR markers from the BALF samples of hospital admitted patients. In this study, downstream analysis of the sequencing data was carried out through the standard Explify analysis (reads based) as well as through CZID seq where NT based NCBI database is used for taxonomic identification. It allowed us to provide consensus for the detected microbial species and also query our data set for extensive findings. Explicitly, mNGS detected microbes (*A. baumannii* (7), *K. pneumoniae*, *S. pneumoniae* and *P. aeruginosa*) even in those patient samples, which reported negative by clinically used BioFire panel. Moreover, several samples (negative on BioFire) showed single infection by other microbes like *S. maltophilia*, *C striatum*, *A. xylosoxidans*, *S. mitis*, *V. parvula* and *P. melaninogenica* warranting unsupervised investigation of microbes for LRTI.

Uniquely, mNGS identified and differentiated mixed infections involving multiple species in several cases, which demonstrated polymicrobial pathogenesis. These observations indicate potential involvement of several bacterial species along with the reported pathogenic ones in determining the differential severity and clinical manifestation of LRTI. The most commonly occurring pathogenic species across all groups of samples included, *A. baumannii, K. pneumoniae*, and *P. aeruginosa*. This observation is similar to other studies, such that of Biagio Santella et al., in which they detected *A. baumannii, S. aureus*, *P. aeruginosa*, and *K. pneumoniae* as the major LRTI-causing microorganisms [[21], [22], [23], [24], [25], [26]].

Interestingly, RPIP-Explify allowed us to capture other inhabitants of lower respiratory tract, which might have affected the diverse clinical severity observed in our LRTI cohort. The common occurrence of low virulence species such as *Rothia mucilaginosa* and *Veillonella parvula* across 25 % of samples deliberates attention for their alleged role in LRT infections. The occurrence of *Veillonella* is closely associated with enrichment of multiple pathogens such as *S. aureus* and *P. melaninogenica*. Further *Veillonella* has been reported as an indicator of dysbiosis following viral infections in URT [27]. Other species like *S. maltophilia* are gaining importance as causal for nosocomial pneumonia due to its characteristic inherent resistance to many broad-spectrum antibiotics including beta-lactams, aminoglycosides and carbapenems [28]. *E. coli* is an uncommon respiratory pathogen but is commonly identified in nosocomial and community-acquired infections [29]. Thus, occurrence of infections by polymicrobial species deliberates the need to tailor treatment strategies for the management of complex respiratory disease effectively.

The prominence of the bacterial species other than the respiratory pathogens captured by mNGS technique was clearly demonstrated for their differential presence in the LRT-WP and WoP patient subgroups. *A. baumannii* has emerged as a major player in patients of LRTI with pneumonia features. Several patients harboured single infections with bacterial species, *S. aureus, S. mitis, S. maltophilia, P. aeruginosa, E. coli* and *R. mucilaginosa* in the LRTI-WP. LRTI-WoP patients also displayed single infections with *P. aeruginosa, S. mitis, V. parvula, P. melaninogenica, C. striatum* and *S. maltophilia*. Generally, *Streptococcus pneumoniae* has been reported as the major cause of pneumonia and found to be present as the most common bacterial pathogen in patients with pneumonia [30,31], but in our study, Explify detected *S. pneumoniae* as one of the unique species associated with the WP patients. Interestingly, CZID seq analysis captured *S. pneumoniae* in a comparatively larger number of patients in both WP and WoP groups. Yet, their presence was >50 % in composition in the majority of the samples, coexisting as polymicrobial infection, which again deliberates the contribution of additional non-respiratory pathogens to the differences observed in the disease severity between the two patient subgroups. Similarly, the unique presence of *C. sputigena* and *K. quasipneumoniae* which have rarely been reported in pneumonia cases can be considered as emerging new infections responsible for increased pneumonia cases in LRTI patients as captured by the mNGS technology. Another case in WP group demonstrated the presence of *Leuconostoc lactis* and *Weissella confuse* by CZID seq analysis with the absence of other respiratory pathogens, in the patient [32]. Reportedly, bacterial coinfections with viral respiratory pathogens also enhance the disease severity in respiratory infections [33]. Contextually, Influenza virus was detected along with pathogenic bacterial species (majorly single infection) in 05 patients of LRTI-WP, which might have augmented the disease course. Contrarily, in WoP LRTI, influenza infection occurred in patients showing multiple bacterial infection, reportedly non-respiratory pathogens.

Microbial resistance patterns were quite prevalent amongst LRTI bacterial infections as evident by the identification of 24 AMR gene families distributed across 09 AMR classes through RPIP-Explify, which extended to 11 classes via CZID seq analysis in WP and WoP patients. Contrarily, BioFire was specialized to capture MRSA and a few important Beta lactams (CTX-M, NDM, VIM, OXA-48, and IMP). *A. baumannii* followed by *K. pneumoniae* showed as highly resistant species through AMR gene profile of LRTI patients. MPH, ABC-F, ANT (3”), SUL, OXA, and 16S RMTase genes in WP group likely originated from *A. baumannii* (MPH, SUL, ABC-F, 16S RMTase), and *K. pneumoniae* (OXA). MDR strains of *A. baumannii* reportedly show resistance to penicillins, cephalosporins, macrolides, tetracyclines, chloramphenicol, quinolones, sulfonamides and trimethoprim [34,35]. The macrolide genes detected in the WP group were *mphE/A/N* whereas carbapenem resistant genes were OXA-278/232/23/9. Alongside, in WoP, CTXM-15 showed the presence in majority of the patients (n = 7) associated with *K. pneumoniae.* The CTX-M-15 gene detected in our WoP group belonging to ESBLs, is one of the most widespread gene among *Enterobacteriaceae*, particularly *K. pneumoniae*, developing carbapenem resistance in clinical samples [36]. Moreover, SHV, TEM, CTX-M, and OXA genes have been shown to be implicated in ESBL (extended spectrum beta-lactamases) phenotype conferring resistance to a broad range of beta-lactam antibiotics such as cephalosporins, penicillin, and monobactam [37]. Few patients also demonstrated the presence of SHV11/1, TEM 1/27 and QnrB/S significantly correlated with *K. pneumoniae* in WoP patients. Clement et al. reported the presence of SHV-11 (41.8 %), TEM (39.5 %), CTX-M-15 (35.3 %), and NDM-1 (6.7 %) genes for *K. pneumoniae* through meta-analysis on published data available from the Asian population [38]. The New Delhi metallo-β-lactamase (NDM) is also an emerging carbapenemase responsible for causing resistance to all cephalosporins, penicillin, and carbapenem antibiotics [39]. The association of NDM 1/2/5/9 was significant for *P. aeruginosa* in few LRTI cases. Deliberating the presence of these AMR genes in our LRTI sample cohort might have enhanced severity and decreased antibiotic treatment options available in LRTI infections. This was in concordance with our findings where the persistence of a greater number of AMR gene families in multiple samples in the WP patients when compared to WoP patients displayed severity of this group, with high ventilator support and mortality rate.

**Fig. 7:**
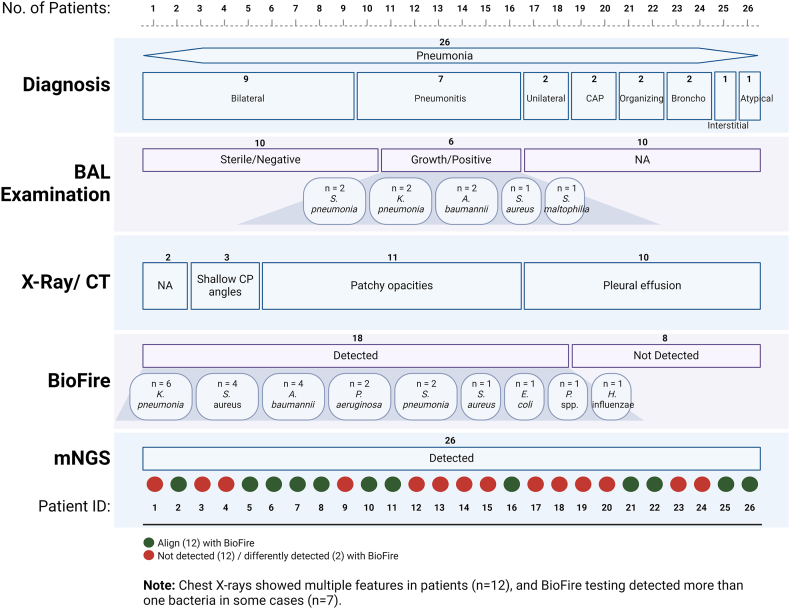
**Clinical data summary of 26 LRTI-WP patients.** In addition to the clinical details provided in the supplementary, it summarizes details toward pneumonia diagnosis, BAL examination, X-ray/CT findings, and pathogen detection via BioFire; its comparison with mNGS data is being presented.

One of the major concerns regarding these studies is its clinical implications and utility. As an attempt, we have summarized in a table the entire clinical and microbiological findings for the patients diagnosed with pneumonia (Additional File 6). To clinically diagnose cases with pneumonia, combination of laboratory (TLC), radiological (X-ray, CT chest) and microbiological findings (culture of urine, blood, Sputum, BAL) are taken into consideration. Based on these clinical findings, these patients had clinically defined Pneumonia, as confirmed by the clinician. Further, we added mNGS findings for each of the patients, which clearly demonstrates presence of pathogenic species in all the cases of pneumonia. Fig. 7 demonstrates summary of the clinical diagnosis of the LRTI-WP patients along with the pathogen identified through BAL culture examination, BioFire and mNGS. For many cases, although BAL culture result came negative with “sterile” or Biofire filmarray showed microbes “not detected”, yet, mNGS was able to identify pathogenic microbes like *A. baumanni*, *P. aeruginosa, K. pneumoniae*, *S. pneumoniae*, *S. maltophilia*, *E. coli* etc. in these patients, reinforcing the diagnosis of Pneumonia. Thus we see, a clear correlation of clinical and microbiological data is demonstrated by our study which strengthens the clinical utility of mNGS. Table 4 compares the detection rates of mNGS and the BioFire panel for each pathogen. Moreover, we observe that many patients were given multiple antibiotics where a guided medication could have been given and vice versa, as well as many patients carried resistance to the genes for which antibiotics were given **(Additional File 6)**.

**Table 4:** Detection rate of different pathogens from Biofire array and mNGS.

Pathogen	Biofire array	mNGS
*Klebsiella pneumoniae*	21.4	35.7
*Acinetobacter calcoaceticus-baumanni complex*	12.9	35.7
*Pseudomonas aeruginosa*	17.1	23.8
*Escherichia coli*	10.0	23.8
*Proteus* spp.	1.4	19.0
*Human Rhinovirus/Enterovirus*	14.3	15.5
*Staphylococcus aureus*	11.4	13.1
*Influenza A*	11.4	13.1
*Streptococcus pneumoniae*	7.1	11.9
*Haemophilus influenzae*	5.7	9.5
*Human Metapneumovirus*	11.4	7.1
*Serratia marcescens*	2.9	2.4
*Respiratory Syncytial Virus*	4.3	1.2
*Parainfluenza Virus*	1.4	1.2

**Fig. 8:**
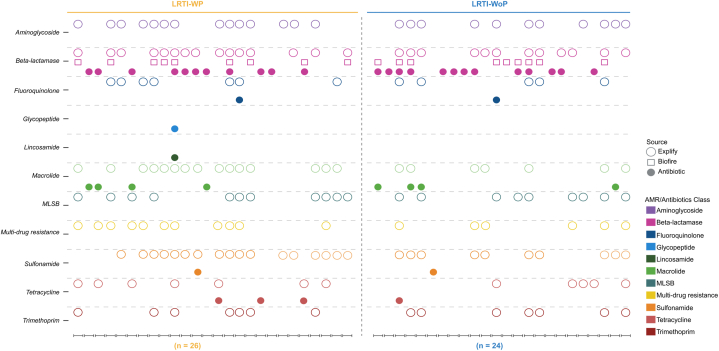
Illustrates the AMR classes detected by the BioFire and Explify methods, along with the antibiotics prescribed to the patients.

We also captured the antibiotic treatment regimen provided to the patients of both groups “WP and WoP” in view of the AMR gene findings from BioFire and Explify (Fig. 8). Incidentally, a higher number of patients in LRTI-WoP group (n = 08) did not carry antibiotic resistance genes owing to the mild polymicrobial presence of infection than WP patients. Moreover, in the WP group, those with Sulfonamide, Tetracycline, Fluoroquinolone, and Beta-lactam resistance received antibiotics from the same AMR class. Conversely, in the WoP group, aside from beta-lactamase, no overlap in antibiotics and AMR classes was observed, potentially contributing to milder cases compared to pneumonia patients. Thus, the incorporation of mNGS technologies into clinical practice might aid in mitigating the substantial majority of AMR burden by detecting and responding to outbreaks promptly.

Thus, the accurate identification of the causative agents of respiratory infections through mNGS can significantly enhance patient management by facilitating timely and effective antibiotic therapy. Appropriate diagnosis can aid in the prevention of secondary infection spread, shorten hospital stays, and ultimately reduce overall healthcare costs.

## 7 Confounding effects and limitations of the study

Heterogeneity across the patient population might act as confounding factor. We addressed this partially in term of age across LRTI WP and LRTI WoP, since age is considered as a criteria during diagnosis of pneumonia. To evaluate the impact of age on our findings, we had performed a Mann-Whitney test comparing patients LRTI WP and LRTI WoP. Our analysis revealed no significant differences (WP- 62.4yrs; WoP- 64.8yrs; p value - 0.96), suggesting that heterogeneity in patients’ age does not affect our categorization of patients into lower respiratory infections with or without pneumonia groups. This adjustment helps mitigate the influence of age on our study outcomes. Additionally, to mitigate the effect of false positives/negatives in mNGS data, careful handling of patient samples was done during library preparation along with stringent and robust analysis pipelines which helps negate occurrence of false positive/negative data.

While our study demonstrates the potential of mNGS in identifying microbial pathogens and associated AMR genes in the BALF samples on a larger scale, we acknowledge the challenges to its practical implementation in everyday clinical settings. Firstly, the turnaround time for mNGS results can be longer compared to traditional diagnostic methods. This delay can be critical in acute clinical scenarios where rapid diagnosis is essential for effective treatment. Secondly, the cost of mNGS remains high, which can be a barrier for widespread adoption in many healthcare facilities, particularly in low- and middle-income countries. The expense is due not only to the sequencing itself but also to the need for specialized equipment and trained personnel. Lastly, the practicality of implementing mNGS in hospitals involves considerations such as the need for robust bioinformatics infrastructure, data storage, and interpretation capabilities. Training healthcare professionals in the use of this technology and ensuring compliance with data privacy regulations are additional challenges. Additionally, potential limitation of the RPIP NGS method, despite its advantages, is a probe-based technique that targets specific species of microbes and AMR genes. This means it may miss pathogens or resistance markeFfilers not included in the probe set. Despite these obstacles, ongoing advancements in sequencing technology, relative cost reduction, and the integration of mNGS into clinical workflows hold promise for its future application. Further research and investment in these areas will be essential to overcoming the barriers to the general use of mNGS in clinical settings.

## 8 Conclusion

This study highlights clinical evaluation and pathogenesis (microbes/AMR) of lower respiratory tract infections in hospital admitted patients from north India. Utilizing the comparative detection potential of BioFire and mNGS, we identified double and polymicrobial infections in multiple patients, which could address different phenotypes and disease severity observed in LRTI patients. Moreover, along with bacterial species, the AMR gene profile too dissociated between the two major groups of LRTI, with and without pneumonia, wherein higher antimicrobial resistance was encountered in the WP group. Henceforth, it is suggestive that mNGS has significant clinical value as it provides early detection of pathogens as well as AMR genes, assisting in better diagnosis of the patients, enabling proper/correct medical treatment regimens.

## Funding

Bill and Melinda Gates Foundation, United Kingdom. [INV-033578]. Rockefeller Foundation, United States (2021 HTH 018).

## Data sharing statement

The datasets generated during the current study are available in the NCBI SRA with the Bio Project accession number PRJNA1083436.

## CRediT authorship contribution statement

**Uzma Shamim:** Writing – original draft, Investigation, Visualization, Methodology, Formal analysis, Validation. **Aanchal Yadav:** Writing – original draft, Investigation, Visualization, Methodology, Formal analysis, Validation. **Ranjeet Maurya:** Writing – original draft, Visualization, Formal analysis, Data Curation, Validation. **Priti Devi:** Writing – original draft, Investigation, Formal analysis, Visualization, Validation. **Pallawi Kumari:** Writing – original draft, Formal analysis, Visualization, Validation. **Kanika:** Writing – original draft, Visualization. **Kriti Khare:** Investigation. **Bansidhar Tarai:** Resources. **Rajesh Pandey:** Writing – review & editing, Supervision, Resources, Project administration, Funding acquisition, Conceptualization.

## Declaration of competing interest

The authors declare that they have no known competing financial interests or personal relationships that could have appeared to influence the work reported in this paper.

## List of abbreviations

LRTI: Lower Respiratory Tract Infection
mNGS: metagenomic next-generation sequencing
AMR: Antimicrobial resistance
RPIP: Respiratory Pathogen ID/AMR
LRTI-WP: Lower Respiratory Tract Infection-With Pneumonia
LRTI-WP: Lower Respiratory Tract Infection-Without Pneumonia
ESBL: Extended Spectrum Beta-Lactamases
